# In-Wheel Motor Fault Diagnosis Using Affinity Propagation Minimum-Distance Discriminant Projection and Weibull-Kernel-Function-Based SVDD

**DOI:** 10.3390/s23084021

**Published:** 2023-04-15

**Authors:** Bingchen Liu, Hongtao Xue, Dianyong Ding, Ning Sun, Peng Chen

**Affiliations:** 1 School of Automotive and Traffic Engineering, Jiangsu University, Zhenjiang 212013, China; 2222004032@stmail.ujs.edu.cn (B.L.);; 2 College of Automotive and Traffic Engineering, Nanjing Forestry University, Nanjing 210037, China; ningsun@njfu.edu.cn; 3 Graduate School of Bioresources, Mie University, 1577 Kurimamachiya-cho, Tsu 514-8507, Japan; chen@bio.mie-u.ac.jp

**Keywords:** in-wheel motor, vibration signal, fault diagnosis, affinity propagation minimum-distance discriminant, multi-class support vector data description, Weibull kernel function

## Abstract

To effectively ensure the operational safety of an electric vehicle with in-wheel motor drive, a novel diagnosis method is proposed to monitor each in-wheel motor fault, the creativity of which lies in two aspects. One aspect is that affinity propagation (AP) is introduced into a minimum-distance discriminant projection (MDP) algorithm to propose a new dimension reduction algorithm, which is defined as APMDP. APMDP not only gathers the intra-class and inter-class information of high-dimensional data but also obtains information on the spatial structure. Another aspect is that multi-class support vector data description (SVDD) is improved using the Weibull kernel function, and its classification judgment rule is modified into a minimum distance from the intra-class cluster center. Finally, in-wheel motors with typical bearing faults are customized to collect vibration signals under four operating conditions, respectively, to verify the effectiveness of the proposed method. The results show that the APMDP’s performance is better than traditional dimension reduction methods, and the divisibility is improved by at least 8.35% over the LDA, MDP, and LPP. A multi-class SVDD classifier based on the Weibull kernel function has high classification accuracy and strong robustness, and the classification accuracies of the in-wheel motor faults in each condition are over 95%, which is higher than the polynomial and Gaussian kernel function.

## 1. Introduction

Faced with the problems of resource shortage and environmental pollution, the development of electric vehicles has become a mainstream trend of the automobile industry, especially electric vehicles with in-wheel motor drive [1]. This is because the in-wheel motor drive technology not only simplifies the components of the vehicle drivetrain and enhances the efficiency of the driving system but also flexibly controls the vehicle and optimizes its spatial arrangement [2,3,4]. At present, some literature exists on the control of an electric vehicle with in-wheel motor drive, such as a fault-tolerant control [5] and a linear parameter-varying controller [6]. The stability and activity of the steering system are improved. In view of the body structure and automobile theory, an even number of in-wheel motors are equally distributed on both sides of the vehicle, and each in-wheel motor is specially installed in the wheel. Moreover, the running condition of the vehicle is frequently varied, and the operation environment is hostile. However, it is also easy to induce damage to some parts of an in-wheel motor. When the damage is severe, some mechanical faults occur. When one or more in-wheel motors go astray, the driving torques on both sides of the body will lose balance. The vehicle is then pulled to one side, which could endanger the security of the vehicle and the driver. Therefore, it is necessary to establish an effective real-time monitoring and fault diagnosis system for the running state of an in-wheel motor.

It is well-known that some pre-processing works, such as noise reduction [7,8] and feature extraction [9,10], can improve the accuracy of fault diagnosis [11,12]. For many interference factors or a complex environment, multidimensional condition monitoring information is often used to extract the object of a follow-on process. However, the response speed of the diagnosis system is reduced to some extent. Then, it is important that high-dimensional condition data are reduced into low-dimensional fault features. At present, dimension reduction algorithms can be divided into two categories, namely, linear and nonlinear dimension reduction [13]. The linear dimension reduction method is based on using a linear transformation to find the appropriate projection direction so as to obtain a low-dimensional observation of the original data. This method is good at discovering the overall characteristics of data, while the nonlinear dimension reduction method involves the expansion of nonlinear data, which is better at exploring the distribution law of complex, nonlinear data. The linear dimension reduction method realizes the mapping of data from a high-dimensional space to low-dimensional space by learning a specific linear transformation. Its essence is to find the basis of a new space to transform the data as a whole [14]. The global structure is a type of important information in the spatial distribution of data, and many classical feature extraction methods are proposed based on this information. The most common methods are the principal component analysis (PCA) [15], linear discriminant analysis (LDA) [16], Fisher discriminant analysis (FDA) [17], etc. The LDA constructs an optimal projection matrix by minimizing intra-class divergence and maximizing inter-class divergence. The LDA can prevent the problems of a long identification time and low classification efficiency due to the relatively high feature dimension [18]. The LDA is a dimensionality reduction algorithm based on the overall sample structure, but it cannot consider the local manifold structure of the sample. The locality-preserving projection (LPP) algorithm is then proposed to linearize the traditional Laplacian feature mapping algorithm to achieve data clustering performance with a smaller computation loss [19]. The LPP algorithm considers the local structure of the sample, but neglects the category information conducive to classification; thus, more information about the spatial structure of high-dimensional data cannot be explored. Based on the advantages and disadvantages of the LDA and LPP algorithms, the minimum-distance discriminant projection (MDP) algorithm is proposed to introduce the weight of inter-class similarity and intra-class similarity for evaluating the distances between all samples and the inter-class and intra-class centers [20]. However, the above methods are only based on the mean value of the whole class, and neither consider the spatial structure distribution of the samples nor think about the nearest neighbor relationship among the different classes. Affinity propagation (AP) is used to cluster large-scale data [21,22]. AP is also employed to improve the traditional MDP algorithm and propose a new dimension reduction algorithm, which is defined as affinity propagation minimum-distance discriminant projection (APMDP). APMDP takes the nearest neighbor relationship and the local structure information of samples as important reference indexes to select the clustering center and construct the sample neighborhood.

Moreover, it is essential to select a suitable and effective classifier for an on-time fault diagnosis system of an in-wheel motor. In recent years, there are many classification algorithms that have been widely used in the field of fault diagnosis, such as the support vector machine [23,24], artificial neural networks [25], convolutional neural network (CNN) [26,27], multi-scale CNN [28], artificial hydrocarbon network [29], generative adversarial network [30], dynamic Bayesian network [31], graph attention network [32], segmentation framework [33], Gaussian process regression [34] and hidden Markov model [35]. These methods are constructive to the fault diagnosis system of an in-wheel motor. However, the traditional fault diagnosis and monitoring methods are mostly used in off-line diagnosis and can be used rarely in the field of on-line diagnosis which conforms to practical engineering applications [36]. In addition, the problem of missing or unsound fault data in actual engineering also increases the difficulty of on-line diagnosis [37]. It is necessary to diagnose the fault state on the basis of the monitoring data in the normal state. Thus, support vector data description (SVDD) is proposed to construct the hypersphere to achieve the classification of target data and non-target data [38]. Traditional SVDD can only give a description of the target data set and ignores the description of different sample classes in the target data set. Based on this, multi-class SVDD is proposed to construct multiple hyperspheres and realize the simultaneous classification and recognition of two or more types of samples [39]. Moreover, multi-class SVDD introduces different kernel functions to map samples from the input space to the high-dimensional feature space. At present, polynomial and Gaussian kernel functions are often used in the multi-class SVDD algorithm [40]. A polynomial kernel function can realize the prediction of the summary by subjectively setting the power number, but it is not suitable for the power number of a large order of magnitude. The Gaussian kernel function can be mapped to infinite dimensions, but its interpretability is poor and it is easy to overfit. In the field of reliability engineering and data-related fitting, Weibull distribution is widely used. For example, the three-parameter Weibull distribution is combined with a neural network (NN) and genetic algorithm (GA) to estimate kernel density [41]. Certainly, three-parameter Weibull distribution can be transformed into two parameters [42]. The scale and shape parameters can be adjusted to map to infinite dimensions and avoid overfitting, and it has a strong sensitivity to similar logarithmic data points and high differentiation [43]. Therefore, the Weibull function is based on constructing a kernel function, which is called the Weibull kernel function, and the Weibull kernel function is then introduced into a multi-class SVDD algorithm.

Vibration signals contain an abundance of information, and vibration signals are often used to monitor the running state and diagnose a fault in the field of fault diagnosis [44,45]. For a mechanical fault of an in-wheel motor, common bearing faults are regarded as a representative of measured vibration signals. Under near-real operating conditions of an electric vehicle, high-dimensional fault features are extracted to represent the different states of the in-wheel motors. APMDP is presented to reduce the dimension of a follow-on process for accelerating the response speed of the diagnosis system in Section 2. Affinity propagation (AP) is introduced into the minimum-distance discriminant projection (MDP) algorithm to propose a new dimension reduction algorithm, which is defined as APMDP. APMDP not only gathers the intra-class and inter-class information of high-dimensional data but also obtains the spatial structure information. Weibull-kernel-function-based SVDD is proposed to improve the classification accuracy and enhance the robustness in Section 3. A multi-class support vector data description (SVDD) is improved using the Weibull kernel function, and its classification judgment rule is modified into the minimum distance from an intra-class cluster center. Section 4 describes the self-made test bench of in-wheel motor and three bearing faults that frequently occur in engineering practice, builds the diagnosis system of an in-wheel motor, demonstrates the recognition capability of the proposed methods, and compares the stability and robustness of the diagnosis system. Finally, conclusions are summarized, and future research is determined.

## 2. Affinity Propagation Minimum-Distance Discriminant Projection

### 2.1. Minimum-Distance Discriminant Projection

Let a high-dimensional sample set *X* be divided into *C* categories. The *D*-dimensional sample set of the *c*th (*c*∈*C*) class is $X^{c}=\left\{x_{i}^{c}|i=1,\;2,\;\cdots ,\;n_{c};\;x_{i}^{c}\in R^{D}\right\}$. Then, the intra-class divergence matrix *S_w_* and inter-class divergence matrix *S_b_* can be defined as follows:

(1) $$S_{w}=\sum_{c=1}^{C}\sum_{i=1}^{n_{c}}\omega_{i}^{c}\left(x_{i}^{c}-m_{X^{c}}\right){\left(x_{i}^{c}-m_{X^{c}}\right)}^{T}$$

(2) $$S_{b}=\sum_{c=1}^{C}\sum_{i=1}^{n_{c}}\sum_{k=1,k\neq c}^{C}b_{i}^{k}\left(x_{i}^{c}-m_{X^{k}}\right){\left(x_{i}^{c}-m_{X^{k}}\right)}^{T}$$

where $x_{i}^{c}$ is the *i*th sample in the *c*th class$,\;\mathrm{and}\;\omega_{i}^{c}$ and $b_{i}^{k}$ express the weight of the similarity of $x_{i}^{c}$ to the class inner center point $m_{X^{c}}$ and the center point $m_{X^{k}}$ of the *k*th class (*k* ≠ *c*), respectively. In general, $m_{X^{c}}$ and $m_{X^{k}}$ can be determined by the sample mean of the corresponding class.

The objective function of the minimum-distance discriminant projection (MDP) is:

(3) $$J_{\mathrm{MDP}}=\underset{a}{\arg\max}\frac{a^{T}S_{b}a}{a^{T}S_{w}a}$$

The eigenvalues of $S_{w}^{-1}S_{b}$ are calculated and sorted by their values, and the eigenvectors $a_{1}$, $a_{2}$, ⋯, $a_{t}$, corresponding to the top *t* eigenvalues, are used to construct the feature matrix of the MDP algorithm $A_{\mathrm{MDP}}=\left[a_{1},\;a_{2},\cdots ,a_{t}\right]$. The sample set of the *c*th class is then projected to obtain the *t*-dimensional feature set, which is expressed as $X^{c}A_{\mathrm{MDP}}$.

### 2.2. Affinity Propagation Minimum-Distance Discriminant Projection

Based on the MDP algorithm, affinity propagation (AP) is employed to propose a new dimension reduction algorithm, which is defined as affinity propagation minimum-distance discriminant projection (APMDP). APMDP takes the nearest neighbor relationship and the local structure information of samples as important reference indexes to select the clustering center and construct the sample neighborhood. This has the advantages of avoiding the setting of the cluster number in advance and taking the original sample points as the final clustering center.

For the sample set of the *c*th class $X^{c}$, the AP algorithm is used to obtain the cluster number and its center point. Here, the cluster number is labeled as $T^{c}$, the *i*th cluster sample set in the *c*th class is defined as $X_{i}^{c}=\left\{x_{ij}^{c}|j=1,\;2,\;\cdots ,\;n_{ci};\;x_{ij}^{c}\in X^{c}\right\}$, and the corresponding center point is $x_{oi}^{c}$. Therefore, the intra-class divergence matrix $S_{w}^{\prime }$ and inter-class divergence matrix $S_{b}^{\prime }$ can be expressed as:

(4) $${S^{\prime }}_{w}=\sum_{c=1}^{C}\sum_{i=1}^{T^{c}}\sum_{j=1}^{n_{ci}}w_{ij}^{c}\left(x_{ij}^{c}-m_{X_{i}^{c}}\right){\left(x_{ij}^{c}-m_{X_{i}^{c}}\right)}^{T}$$

(5) $${S^{\prime }}_{b}=\sum_{c=1}^{C}\sum_{i=1}^{T^{c}}\sum_{j=1}^{n_{ci}}\sum_{k\neq c}^{C}b_{ij}^{c,k}\left(x_{ij}^{c}-m_{X_{i}^{k}}\right){\left(x_{i}^{c}-m_{X_{i}^{k}}\right)}^{T}$$

where $m_{X_{i}^{c}}$ is the intra-class local mean that is the mean value of $X_{i}^{c}$,$\;m_{X_{i}^{k}}$ is the inter-class local mean that is the mean value of $X_{i}^{k}$ (*k* ≠ *c*), $w_{ij}^{c}$ is the similarity weight between $m_{X_{i}^{c}}$ and each sample $x_{ij}^{c}$ in $X_{i}^{c}$, and $b_{ij}^{c,\;k}$ is the similarity weight between $m_{X_{i}^{k}}$ and each sample $x_{ij}^{c}$ in $X_{i}^{c}$. $w_{ij}^{c}$ and $b_{ij}^{c,\;k}$ can be defined as follows:

(6) $$\omega_{ij}^{c}=\exp\left(-\frac{1}{t}{\left\|x_{i}^{c}-m_{X_{j}^{c}}\right\|}^{2}\right)$$

(7) $$b_{ij}^{c,k}=\left\{\begin{array}{l} \delta ,\delta >\sigma \\ 0,\;\mathrm{others} \end{array}\right.$$

where $\delta =\exp\left(\frac{1}{t}\|x_{ij}^{c}-m_{X_{i}^{c}}\|{}^{2}-\|x_{ij}^{c}-m_{X_{i}^{k}}\|{}^{2}\right)$, *t* is the square of the mean Euclidean distance among all samples, *σ* is adjustable parameter (0 < *σ* < 1).

Similarly, the objective function of APMDP is defined as

(8) $$J_{\mathrm{APMDP}}=\underset{a^{\prime }}{\arg\max}\frac{{a^{\prime }}^{T}S_{b}^{\prime }a^{\prime }}{{a^{\prime }}^{T}S_{w}^{\prime }a^{\prime }}$$

**Figure 1:**
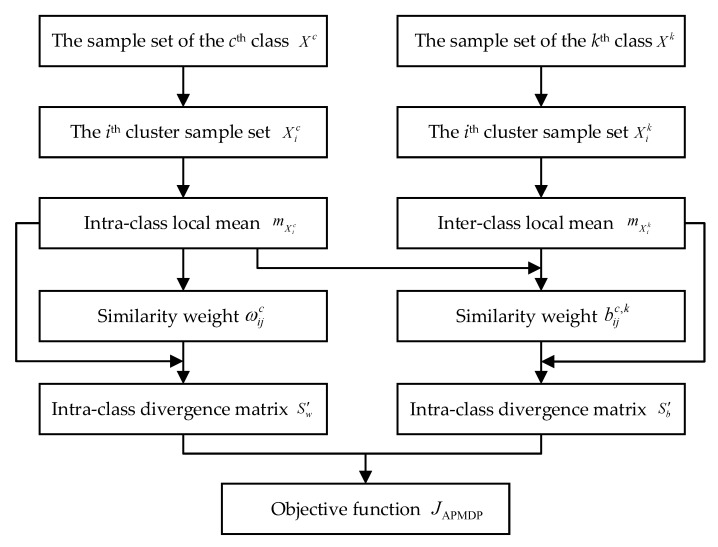
Flow chart of APMDP algorithm.

The APMDP algorithm is shown as Figure 1.

The eigenvalues of ${\left(S_{w}^{\prime }\right)}^{-1}S_{b}^{\prime }$ are calculated to select the top *t* eigenvalues, and the corresponding eigenvectors $a_{1}^{\prime }$, $a_{2}^{\prime }$, ⋯, $a_{t}^{\prime }$, are then confirmed to construct the feature matrix of APMDP $A_{\mathrm{APMDP}}=\left[a_{1}^{\prime },\;a_{2}^{\prime },\;\cdots ,\;a_{t}^{\prime }\right]$. Let $Y^{c}$ be the main projection matrix that the sample set $X^{c}$ has been projected; then, $Y^{c}=X^{c}A_{\mathrm{APMDP}}$. Obviously, $Y^{c}$ is the *t*-dimensional features set that is expressed as $Y^{c}=\left\{y_{i}^{c}|i=1,2,\;\cdots ,\;n_{c};\;y_{i}^{c}\in R^{t}\right\}.$ The sample set *X* is then projected by APMDP to obtain the main projection matrix *Y*.

## 3. Weibull-Kernel-Based SVDD

### 3.1. AP-Based SVDD

SVDD is a promising and popular method for one-class classification or data description. It features a non-parametric sparse model without requiring the knowledge of explicit data distribution [46]. Multi-class SVDD is a generalized SVDD algorithm which finds multiple spheres around the multi-class data [47]. For the main projection matrix *Y*, multi-class SVDD is suitable for the classification of *C* categories samples. When the main projection matrix *Y* is taken as the input, and each type of main projection matrix *Y^c^* is described as a closed and compact hypersphere, and the sample points of *Y^c^* are fully contained or contained or as much as possible in the sphere. The objective function of SVDD is:

(9) minrcrc2+pc∑i=1ncξics.t yic−sc2≤rc2+ξic, ξic≥0

where ***s^c^*** and *r^c^* are the center and radius of the *c*th class hypersphere, respectively, $\xi_{i}^{c}$ is slack variable, *p^c^* is penalty parameter. *K*(*y_i_*, *y_j_*) is introduced instead of the inner product, and the dual form of Equation (9) can be converted into:

(10) $$\begin{array}{c} \max L=\sum_{i=1}^{n_{c}}\alpha_{i}^{c}K\left(y_{i}^{c},y_{i}^{c}\right)-\sum_{i=1}^{n_{c}}\sum_{j=1}^{n_{c}}\alpha_{i}^{c}\alpha_{j}^{c}K\left(y_{i}^{c},y_{i}^{c}\right)\\ s.t\;\sum_{i=1}^{n_{c}}\alpha_{i}^{c}=1,\;0\leq \alpha_{i}^{c}\leq p^{c},\;i=1,2,\ldots ,n_{c} \end{array}$$

The quadratic programming problem corresponding to Equation (10) is solved to obtained *C* hyperspheres, and the center and radius of the *c*th class hypersphere are expressed as:

(11) $$\begin{array}{l} s^{c}=\sum_{i=1}^{n_{c}}\sum_{j=1}^{n_{c}}\alpha_{i}^{c}\alpha_{j}^{c}K\left(y_{i}^{c},y_{j}^{c}\right)\\ {\left(r^{c}\right)}^{2}=K\left(y^{*}{}^{c},y^{*}{}^{c}\right)-2\sum_{i}^{n_{c}}\alpha_{i}^{c}K\left(y^{*}{}^{c},y_{i}^{c}\right)+\sum_{i=1}^{n_{c}}\sum_{j=1}^{n_{c}}\alpha_{i}^{c}\alpha_{j}^{c}K\left(y_{i}^{c},y_{j}^{c}\right)\\ \forall \;y^{*}{}^{c}\in SV^{c} \end{array}$$

For a certain test point *z*, APMDP is used to obtain the corresponding input $z^{\prime }=z\cdot A_{\mathrm{APMDP}}$, and then the distance between the test point *z* and the center *s^c^* of the hypersphere is calculated as follows:

(12) $${\left(d^{c}\right)}^{2}={\left\|z^{\prime }-s^{c}\right\|}^{2}={\left\|z\cdot A_{APMDP}-s^{c}\right\|}^{2}$$

The traditional method is to compare the size relation of *r^c^* and *d^c^*. If $d^{c}\leq r^{c}$, then the test point belongs to the *c*th class [48]. However, it often occurs that multiple hyperspheres are mixed together, and if *d^c^* is smaller than the radius of these hyperspheres at the same time, the simple comparison method is unreliable. Therefore, the AP clustering algorithm is used to optimize the classification criterion of SVDD. In the paper, the classification model is called AP-based SVDD.

The major objective of AP-based SVDD is to refine the distances between the test point and each cluster center of the overlapped hyperspheres to find the cluster and its center with the smallest distance. The test point is then classified correctly. Suppose *l* (*l* ≥ 2) hyperspheres are mixed together. The corresponding classes are labeled with *q*_1_, *q*_2_, …, *q_j_*, …, *q_l_*, the center point of the sample set of the *i*th ($i\leq T^{q_{j}}$) cluster $X_{i}^{q_{j}}$ in the *j*th overlapped hypersphere is $x_{oi}^{q_{j}}$, and the center point after the projection is $y_{oi}^{q_{j}}$. The distance between the test point *z* and the cluster center $y_{oi}^{q_{j}}$ is then calculated by:

(13) $${\left(d_{i}^{q_{j}}\right)}^{2}={\left\|z^{\prime }-y_{oi}^{q_{j}}\right\|}^{2}={\left\|z\cdot A_{APMDP}-y_{oi}^{q_{j}}\right\|}^{2}$$

The traditional minimum distance classification method is optimized to propose a novel criterion which the distance from a cluster center in each intra-class is a minimum. For the test point ***z*** and the cluster center $y_{oi}^{q_{j}}$, the classification criterion of AP-based SVDD can be expressed as follows

(14) $$d_{i^{*}}^{q^{*}}=\underset{q_{j},\;i}{\min}(d_{1}^{q_{1}},d_{2}^{q_{1}},\cdot \cdot \cdot ,d_{T^{q_{{}_{1}}}}^{q_{1}},d_{1}^{q_{2}},d_{2}^{q_{2}},\cdot \cdot \cdot ,d_{T^{q_{{}_{2}}}}^{q_{2}},\cdot \cdot \cdot ,d_{1}^{q_{l}},d_{2}^{q_{l}},\cdot \cdot \cdot ,d_{T^{q_{{}_{l}}}}^{q_{l}})$$

### 3.2. Weibull Kernel Function

SVDD can convert the nonlinear problem in the original space into the linear problem in the higher dimensional space; this is attributed to the kernel function. At present, the polynomial and Gaussian kernel functions are often used in the SVDD algorithm. However, there are two issues that are the focus of the application for the above kernel function. One is the problem of overfitting or parameter selection in a high-order condition, and the other is that a kernel function is used to describe multiple distribution types of observation data. For multiple faults of an in-wheel motor, the monitoring data are distributed differently, and the data distributions in the different operating conditions vary greatly. Then the polynomial and Gauss kernel functions are not a good fit for the diagnosis field of an in-wheel motor fault.

The Weibull function, as the theoretical basis for reliability analysis and life test, is widely used in the data processing of cumulative wear failures and the life testing of various mechanical and electrical equipment [49]. It can not only describe the distribution characteristics of various data but can also distinguish the data with close distances or a high feature similarity. Therefore, Weibull function is used to construct a Weibull kernel function, as follows:

(15) $$K\left(y_{i},y_{j}\right)=\exp\left(-{\left(\frac{\left\|y_{i}-y_{j}\right\|}{\beta }\right)}^{\gamma }\right)$$

where *β* (*β* > 0) is the scale parameter, and *γ* (*γ* > 0) is the shape parameter. Equation (15) is used to obtain the kernel function matrix, and it is then easily proven that the matrix is a symmetric matrix with diagonal elements of 1 and is positive semi-definite. Therefore, if the Weibull kernel function satisfies Mercer’s theorem, it is an effective kernel function.

**Figure 2:**
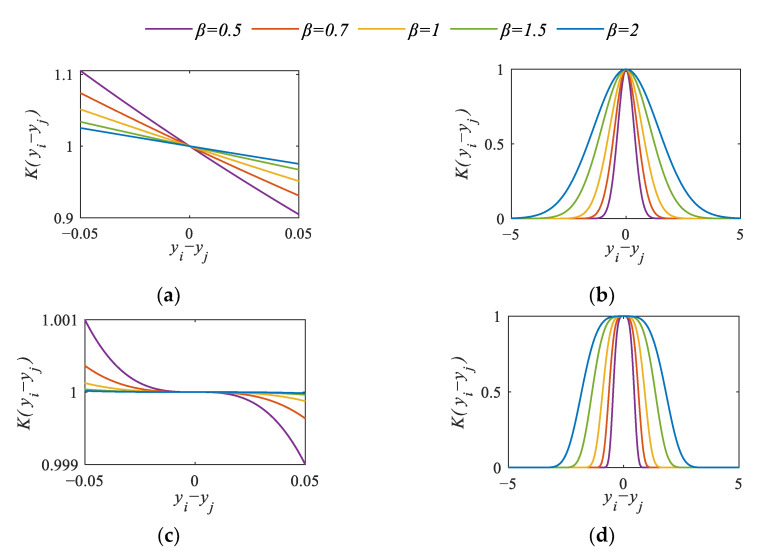
The curves of Weibull kernel function with different parameters: (**a**) *γ* = 1; (**b**) *γ* = 2; (**c**) *γ* = 3; (**d**) *γ* = 4.

Figure 2 shows the curves of a Weibull kernel function with different parameters. In the case of *γ* = 1 or *γ* = 3, the value of *K*(*y_i_*, *y_j_*) decreases with the increase in *y_i_* − *y_j_*. The larger the *β* is, the gentler the curve is. When *γ* = 2 or *γ* = 4, the kernel function has symmetry, the axis of symmetry is *y_i_* − *y_j_* = 0, and the curve becomes more gentle with the increase in *β*. Obviously, the Weibull kernel function is a global kernel function when *γ* is an odd number, and has global characteristics and can extract the global characteristics of samples. When *γ* is an even number, it is a local kernel function that has local features and can extract the local features of the sample. In particular, the Weibull kernel function becomes the Gaussian kernel function when *γ* = 2’ it is obvious that the Gaussian kernel function is a special case of the Weibull kernel function. Therefore, the Weibull kernel function has broader application prospects. The appropriate parameters are selected to describe uniformly different distribution types of data.

### 3.3. Weibull-Kernel-Function-Based SVDD

The Weibull kernel function was selected to calculate the center and radius of each class hypersphere. Then, the objective of SVDD was converged for multi-class classification. In this paper, the algorithm is called Weibull-kernel-function-based SVDD. To verify the robustness and effectiveness of the proposed method, three data sets were selected by the University of California, Irvine (UCI), and SVDD algorithms based on polynomial, Gaussian, and Weibull kernel functions were then performed. The recognition accuracies and running times were analyzed as shown in Table 1. However, it is important to note that all data were directly input into different SVDD algorithms for verifying the performance of each kernel function.

On the whole, the recognition accuracy of Weibull-kernel-function-based SVDD is higher than the polynomial and Gaussian kernel functions, and the running time is also slightly less. According to the local situation analysis, there are many differences in the discriminations of the three data sets. In particular, the features of the three classes in the Seeds data set are ambiguous, the recognition accuracies of the SVDD based on polynomial and Gaussian kernel functions are generally low, and one of Class II is not more than 50%. However, the Weibull-kernel-function-based SVDD can boost the recognition accuracy by up to 80%. Therefore, the Weibull-kernel-function-based SVDD has strong robustness and high classification ability.

## 4. Experiment Verification

To verify the effectiveness of the proposed fault diagnosis system using APMDP and the Weibull-kernel-function-based SVDD, in-wheel motor monitoring data from the self-made test stand were studied in this section. Moreover, traditional LDA, MDP, and LPP are performed with the experimental data, and the classification performances of SVDDs based on different kernel functions are compared.

### 4.1. In-Wheel Motor Test Bench

**Figure 3:**
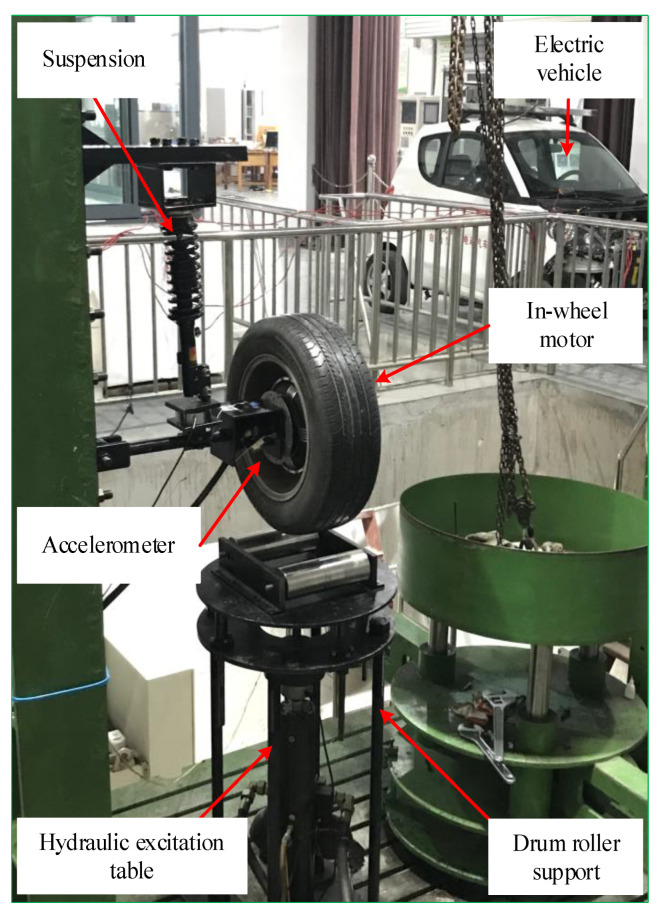
In-wheel motor test bench.

Figure 3 is a photograph of the in-wheel motor test bench that was designed to simulate the actual operating condition of an electric vehicle. The installation of the in-wheel motor was the same as real electric vehicle, which has a stator axis mounted on the suspension and the rotor fixed into the hub of a wheel. A hydraulic excitation table was controlled to simulate road shocks, and the base height of the table was adjusted to simulate a vertical load of 1 ton. The power supply and controller of the real electric vehicle were used directly to control the rotating speed of the in-wheel motor, and a digital tachometer (Type: DM6234P) was used to measure the speed so that the speed was stable in 300, 400, 500, or 600 rev/min and thereabouts. The accelerometer (Type: QKC100LTH; Sensitivity: 13.3 mV/A) was fixed on the stator axis to acquire the vibration signal. Moreover, the features of bearing faults, the processing power, and the timeliness of the on-board processor were considered, the sampling frequency was set to 12.8 kHz, and the sampling time was chosen at 60 s. Vibration signals were collected by an LMS SCADAS mobile multifunctional data acquisition instrument and its supporting LMS Test-Lab Rev 11B software.

**Figure 4:**
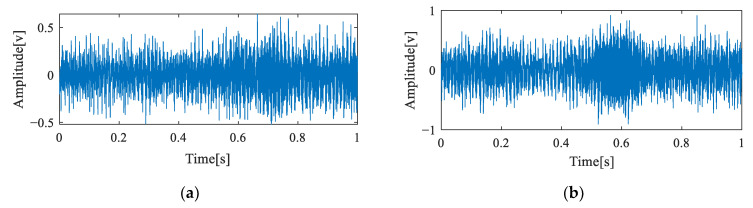

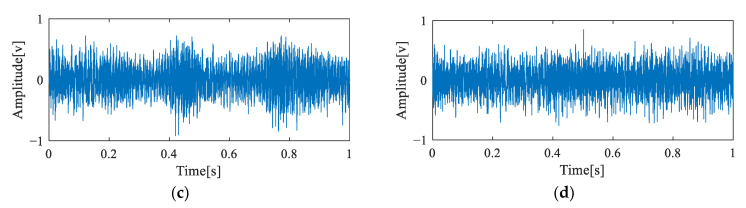
Original vibration signals of in-wheel motors with different states: (**a**) normal state; (**b**) inner race fault; (**c**) rolling element fault; (**d**) outer race fault.

To quantitatively research the faults of the in-wheel motor, three common bearing faults such as an inner race defect, rolling element defect, and outer race defect were artificially built onto the stator axis of each in-wheel motor by professionals. Certainly, the dimensions of the in-wheel motor were DU2505237, and the defect sizes were the same: the width was 0.3 mm, and the depth was 0.1 mm. In the process of performing the experiments, a normal in-wheel motor was first used to demarcate the basic operating environment and the control voltages for different speeds. In-wheel motors with an inner race fault, rolling element fault, and outer race fault were exchanged in order to run in the above controlled and operated environment. The partial experiment signals of four different in-wheel motors, including a normal state and three faults at 600 rev/min, are shown as in Figure 4.

### 4.2. Construction of In-Wheel Motor Fault Diagnosis System

Firstly, symptom parameters (SPs) were extracted from vibration signals to represent the different states of the in-wheel motor. Since the rotating speed of an in-wheel motor changes frequently and the operating environment is complicated, it is difficult to select SPs in single domain to distinguish multiple faults [50]. Eight SPs in time and frequency domains, as shown in Table 2, were then chosen to construct an observation sample of the in-wheel motor in each condition.

It is well-known that an observation period contains at least two cycles of sampling points; thus, 8192 sampling points were taken as an observation sample in this study. The original vibration signal from the in-wheel motor in each condition was filtered by a hand-pass filter with 1~5 kHz to calculate the corresponding SPs and form the SP set {*P*_1_, *P*_2_, …, *P*_8_} of an observation sample. Therefore, 90 observation samples could be obtained for each condition of the in-wheel motor; of them, 60% were used as training samples, and the others were used as test samples.

Secondly, APMDP was used to refine the in-wheel motor observation samples into a lower-dimensional features set, which was regarded as the input of the SVDD model. In view of the different states of the in-wheel motor, with its varied rotating speed and complex operating environment, the target of the dimension reduction was set as three, and the features after the APMDP were recorded as the first, second, and third projections, in order. Meanwhile, the update step of the adjustable parameter *σ* was set to 0.1, and the optimal value was 0.7, based on the experimental data. When the training samples of the in-wheel motor with four states in the same rotating speed were input into the APMDP algorithm, the feature matrix corresponding to the rotating speed *A*_APMDP_ can be established, and then the three-dimensional feature set of the in-wheel motor was obtained. Here, the feature matrix ***A*_APMDP_** of the in-wheel motor t 300 rev/min is shown as follows:

$$A_{APMDP}={\left[\begin{array}{ccc} -0.0153 & 0.0050 & -0.0111\\ \cdots & \cdots & \cdots \\ 0.2050 & -0.2807 & 0.5198\\ -0.9786 & -0.9598 & -0.8542 \end{array}\right]}_{8\times 3}$$

Finally, the three-dimensional feature set of the in-wheel motor was used to build the fault diagnosis model of the Weibull-kernel-function-based SVD. In the study, a differential evolution algorithm [51,52] was utilized to optimize the parameters of the in-wheel motor diagnosis system, such as the scale parameter *β* and the shape parameter *γ* of the Weibull kernel function, and the punitive coefficient *p* of the multi-class SVDD model. The population size was then set to 100, the evolution algebra to 50, the mutation operator to 0.5, and the crossover operator to 0.2. For clear expression, rotating speeds of 300, 400, 500, or 600 rev/min and thereabouts are denoted as the operating conditions 1, 2, 3, and 4 of the in-wheel motor, respectively, and the parameters *β*, *γ*, and *p* of the in-wheel motor diagnosis system are given the numeric subscript of *i* (*i* = 1, 2, 3, and 4), such as, *β_i_*, *γ_i_*, and *p_i_*; therefore, the values 1, 2, 3, and 4 stand for the normal state, inner race fault, rolling element fault, and outer race fault, respectively. When the three-dimensional feature sets of the in-wheel motor with four states in the same condition were input into the algorithm of the Weibull-kernel-function-based SVDD, the parameters of the in-wheel motor diagnosis system were confirmed. Here, the diagnosis model of the in-wheel motor in operation condition 1 is introduced as an example: the scale and shape parameters of Weibull kernel function corresponding to the four in-wheel motor four states are *β*_1_ = 6.246, *β*_2_ = 2.171, *β*_3_ = 5.748, *β*_4_ = 1.467, *γ*_1_ = 4, *γ*_2_ = 8, *γ*_3_ = 10, and *γ*_4_ = 6, respectively, and the punitive coefficients of the multi-class SVDD model corresponding to the four in-wheel motor states are *p*_1_ = 0.747, *p*_2_ = 0.859, *p*_3_ = 0.646, and *p*_4_ = 0.959, respectively. In this way, the diagnosis models of the in-wheel motor in operation conditions 2, 3, and 4 were built, and thus the fault diagnosis system of the in-wheel motor was determined.

### 4.3. Construction of In-Wheel Motor Fault Diagnosis System

**Figure 5:**
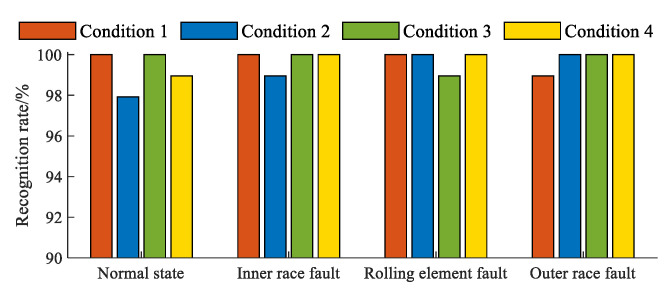
Diagnosis result of each in-wheel motor fault.

To verify the effectiveness of the proposed method, the unused observation samples from the above experiment were planned to evaluate the fault diagnosis system of the Weibull-kernel-function-based SVDD. Firstly, the above feature matrix *A*_APMDP_ of the in-wheel motor in each condition was directly used to refine the observation samples into the three-dimensional feature set. Secondly, the three-dimensional feature set of the in-wheel motor in each condition was input into the fault diagnosis system, and the diagnosis states of the in-wheel motor were then obtained. According to the actual state of each observation sample, the recognition rates of each in-wheel motor state in different condition were counted, as shown in Figure 5. It is obvious that all recognition rates are over 98.75% and most of them remain above 99%, meaning that the proposed diagnosis system can be applied to recognize the common bearing faults of an in-wheel motor.

**Figure 6:**
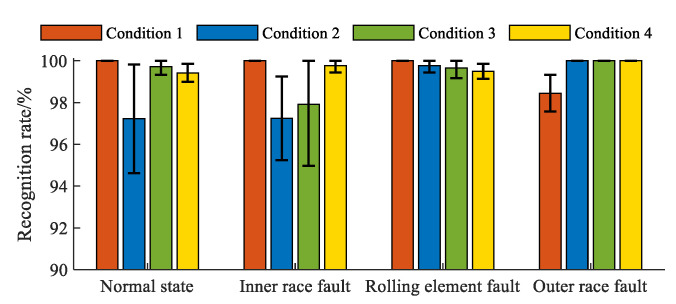
Average diagnosis result of each in-wheel motor fault with different training options.

Moreover, different numbers of observation samples were planned as training samples to demonstrate the robustness of the proposed diagnosis system. The above option of training samples was used to plan the other two. One option is that the observation samples are equally divided into training and test samples; another is to select 40% of the observation samples for training the diagnosis model and use the others for testing the ability. The above parameters of the in-wheel motor diagnosis system remain constant, and each diagnosis model is built to recognize the corresponding test samples for each condition. The classification accuracies of the four states of the in-wheel motor for each condition were over 95%. Here, the average accuracies of the three diagnosis models with different training options are shown in Figure 6. Although the training samples have significant differences, the fluctuations in the diagnosis accuracies are small. This indicates that the proposed diagnosis system for the in-wheel motor has good robustness. 

### 4.4. Comparison with Other Methods

To further confirm the contribution of the proposed diagnosis method for the in-wheel motor, APMDP and Weibull-kernel-function-based SVDD were compared with other methods on the basis of the same experimental data above.

Firstly, the performance of APMDP was analyzed using a divisibility parameter and compared with popular dimension reduction methods such as LDA, MDP, and LPP. Certainly, there are different definitions of divisibility parameter, one of which is based on the distances between the inter-class and intra-class, which considers not only the different inter-class influence factors but also the extent of the intra-class influence [53]. Let $y_{i}^{c}\;\left(c=1,\;2,\;\cdots ,\;C;i=1,2,\;\cdots ,\;n_{c}\right)$ be the element of the lower-dimensional features set, and $\bar{y_{c}}=\frac{1}{n_{c}}\sum_{i=1}^{n_{c}}y_{i}^{n_{c}}$ is the clustering center of the *c*th class. Then, the divisibility parameter, based on the inter-class distance and intra-class distance, is defined as follows:

(16) $$\rho =\frac{H_{b}}{H_{w}}$$

(17) $$H_{b}=\frac{2}{C\left(C-1\right)}\sum_{c=1}^{C-1}\sum_{s=c+1}^{C}\left\|{\bar{y}}_{c}-{\bar{y}}_{s}\right\|$$

(18) $$H_{w}=\frac{1}{C}\sum_{c=1}^{C}\left(\frac{1}{n_{c}}\sum_{i=1}^{n_{c}}\left\|y_{i}^{c}-{\bar{y}}_{c}\right\|\right)$$

where *H_b_* is the inter-class distance, which reflects the dispersion degree among different classes, and *H_w_* is intra-class distance, which informs the aggregation degree of each class. Obviously, the greater the inter-class distance *H_b_* and the smaller the intra-class distance *H_w_*, the greater the divisibility parameter ρ. This means that the separability of the feature set is better.

For the same experimental data in each condition, LDA, MDP, LPP and APMDP were performed to obtain the lower-dimensional features set and then calculate the divisibility parameters of the four in-wheel motor states, as shown in Table 3. It is obvious that the APMDP-based divisibility parameter for each condition is larger than the other methods, and the divisibility of the in-wheel motor faults is improved by at least 8.35%; thus, APMDP is an excellent dimension reduction method.

**Figure 7:**
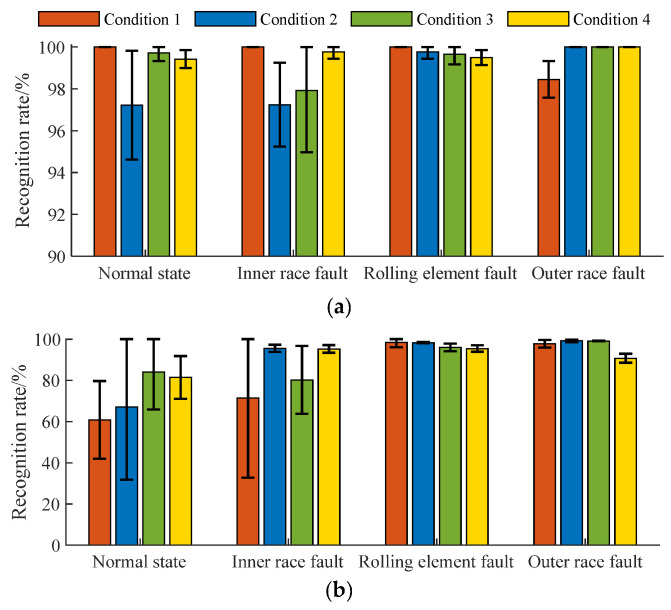
Average diagnosis accuracy of each in-wheel motor fault by multi-class SVDD based on other kernel functions: (**a**) polynomial kernel function; (**b**) Gaussian kernel function.

Secondly, the multi-class SVDD classifiers based on polynomial and Gaussian kernel functions were built to explore the influence of different kernel functions on SVDD performance. Certainly, the basic parameters of algorithm optimization, such as the population size, the evolution algebra, the mutation operator, and the crossover operator, remained constant, and the corresponding training and test samples were used, respectively. Here, 60% of experimental data of the four in-wheel motor states in operation condition 1 were used to train the parameters of the diagnosis system. The optimal orders of the polynomial kernel function corresponding to the four in-wheel motor states were *de*_1_ = 6, *de*_2_ = 6, *de*_3_ = 2, and *de*_4_ = 6, and the corresponding punitive coefficients were *p*_1_ = 0.272, *p*_2_ = 0.772, *p*_3_ = 0.663, and *p*_4_ = 0.289, respectively. Then, the recognition rates of the four in-wheel motor states were 94.79%, 93.75%, 55.21%, and 96.88% respectively. Similarly, the dimension parameters of the Gaussian kernel function were *τ*_1_ = 44.056, *τ*_2_ = 457.542, *τ*_3_ = 502.144, and *τ*_4_ = 508.626; the penalty coefficient of the multi-class SVDD model was *p*_1_ = 0.644, *p*_2_ = 0.895, *p*_3_ = 0.363, and *p*_4_ = 0.472. Then, the recognition rates of the four running states of the wheel motor were 87.50%, 83.33%, 58.33%, and 79.17%, respectively. Similarly, 60% of the experimental data of the four in-wheel motor states in the operation conditions 2, 3, 4, and 50% and 40% of the experimental data were used to build the diagnosis systems based on polynomial and Gaussian kernel functions. Then, the corresponding experimental data were used to test the classification abilities, respectively. Here, the recognition rates of the three diagnosis models with different training options in the same condition are averaged, as shown in Figure 7. Obviously, the stability and generalization of the Weibull-kernel- function-based SVDD are superior to the polynomial and Gaussian kernel functions.

## 5. Conclusions

To effectively monitor the operating state of an in-wheel motor and diagnose the common faults, a novel method using APMDP and a Weibull-kernel-function-based SVDD was proposed to build the fault diagnosis system for an in-wheel motor, and the effectiveness was demonstrated experimentally. The superiority of the methods proposed in the paper can be described by the following points:

(1) The proposed APMDP not only gathered the intra-class and inter-class information of high-dimensional data but also obtained the information about the spatial structure. This is attributed primarily to the reasonable combination of the AP clustering algorithm and traditional MDP algorithm. Specifically, the divisibility of in-wheel motor faults by APMDP was improved by at least 8.35% over LDA, MDP, and LPP.
(2) A multi-class SVDD classifier based on the Weibull kernel function has a high classification accuracy and strong robustness, which essentially results from the Weibull kernel function and the category judgment rule of the minimum distance from the intra-class cluster center in the multi-class SVDD algorithm. Specifically, the classification accuracies of in-wheel motor faults in each condition were over 95%.
(3) The fault diagnosis system using APMDP and Weibull kernel function SVDD was not only adapted to the multi-speed operating conditions of an in-wheel motor but also performed classification with high precision, which is conducive to constructing an on-line condition monitoring system of the distributed drive system based on multiple in-wheel motors.

Considering the actual operating conditions and environment of an electric vehicle, an experiment involving an in-wheel motor with more mechanical and electrical faults under the actual operating conditions and different loads of the electric vehicle will be planned to verify the effectiveness and generalization capability of the proposed methods and subsequently expand their range of application.

## Figures and Tables

**Table 1:** Performance comparison of SVDD algorithms based on different kernel functions.

Data Set		Polynomial Kernel		Gaussian Kernel		Weibull Kernel	
Name	Class	Accuracy/%	Time/ms	Accuracy/%	Time/ms	Accuracy/%	Time/ms
Iris	I	100.0	367	100.0	379	100.0	350
	II	75.0		75.0		75.0	
	III	90.0		90.0		95.0	
Wine	I	86.7	365	86.7	360	86.7	355
	II	73.3		66.7		73.3	
	III	100.0		100.0		100.0	
Seeds	I	55.0	396	55.0	397	65.0	389
	II	45.0		40.0		80.0	
	III	85.0		85.0		85.0	

**Table 2:** Symptom parameters used in an in-wheel motor diagnosis system.

Domain	Features	Definition
Time	Root mean square (RMS)	$P_{1}=\sqrt{\frac{1}{N}\sum_{i=1}^{N}x_{i}^{2}}$
	Mean of Peaks	$P_{2}=E(\left|x_{pi}\right|)$
	Skewness of maximum	$P_{3}=E[{\left(\frac{x_{pi}-\mu_{p}}{\sigma_{p}}\right)}^{3}]$
	Kurtosis of maximum	$P_{4}=E[{\left(\frac{x_{pi}-\mu_{p}}{\sigma_{p}}\right)}^{4}]$
Frequency	Spectral Skewness	$P_{5}=\frac{1}{\sigma^{3}\cdot I}\sum_{i=1}^{I}{\left(f_{i}-\bar{f}\right)}^{3}\cdot F(f_{i})$
	Spectral Kurtosis	$P_{6}=\frac{1}{\sigma^{4}\cdot I}\sum_{i=1}^{I}{\left(f_{i}-\bar{f}\right)}^{4}\cdot F(f_{i})$
	Total power spectrum	$P_{7}=\sum_{i=1}^{I}F(f_{i})$
	RMS of power spectrum	$P_{8}=\sqrt{\frac{1}{I}\sum_{i=1}^{I}F^{2}(f_{i})}$

**Table 3:** Divisibility parameter of four in-wheel motor states by different methods.

Method	Divisibility Parameters of Four In-Wheel Motor States			
	Condition 1	Condition 2	Condition 3	Condition 4
LDA	0.1182	0.6827	0.1998	0.1580
MDP	0.3462	0.2272	0.6220	0.0726
LPP	0.3654	0.7139	0.2859	0.2733
APMDP	0.3959	0.9411	1.0544	0.3783

## Data Availability

Not applicable.
